# Prediction of early postoperative hypocalcemia and hypoparathyroidism after thyroidectomy for papillary thyroid carcinoma: dual-outcome prediction models

**DOI:** 10.3389/fendo.2026.1887201

**Published:** 2026-09-30

**Authors:** Cuiping Zhou, Qian Chen, Shuyan Liu, Nuoxuan Niu, Xinyu Ban, Shiyang Wang, Jinfeng Yan, Ying Bi, Jing Liu, Jie Liu, Yanxia Bai

**Affiliations:** Department of Otorhinolaryngology Head and Neck Surgery, The First Affiliated Hospital of Xi’an Jiaotong University, Xi’an, Shaanxi, China

**Keywords:** hypocalcemia, hypoparathyroidism, nomogram, papillary thyroid carcinoma, preoperative risk stratification, total thyroidectomy

## Abstract

**Background:**

Postoperative hypocalcemia and hypoparathyroidism are two common complications after total or subtotal/near-total for papillary thyroid carcinoma (PTC). This study aimed to identify significant predictors of early postoperative hypocalcemia and hypoparathyroidism and develop prediction nomograms for risk stratification in PTC patients.

**Methods:**

A total of 1036 PTC patients who underwent total or subtotal/near-total thyroidectomy were retrospectively enrolled. Multivariable logistic regression with prespecified variables was performed to identify independent predictors for two outcomes: (1) early postoperative hypocalcemia, and (2) hypoparathyroidism. Nomograms were constructed for each outcome. Model performance was assessed by area under the receiver operating characteristic curve (AUC), calibration plots and decision curve analysis (DCA). Bootstrap internal validation (500 resamples) was used to correct for optimism.

**Results:**

Among 1,036 patients, 325 (31.4%) developed early postoperative hypocalcemia, and 228 (22.0%) met criteria for hypoparathyroidism. For hypocalcemia, independent predictors were preoperative calcium (per 0.1 mmol/L: OR 0.298, 95% CI 0.234-0.374, P < 0.001), thyroidectomy extent (subtotal vs. total: OR 0.75, 95% CI 0.025-0.709, P = 0.033), lateral neck dissection (LND) (OR 3.197, 95% CI 1.742-5.852, P < 0.001), and ultrasound risk score (central lymph node involvement: OR 4.013, 95% CI 2.559-6.323, P < 0.001; lateral lymph node involvement: OR 3.387, 95% CI 1.700-6.825, P < 0.001; primary nodule only: OR 1.899, 95% CI 1.119-3.198, P = 0.016). For hypoparathyroidism, independent predictors were preoperative calcium (per 0.1 mmol/L: OR 0.417, 95% CI 0.330-0.519, P < 0.001) and ultrasound risk score (central lymph node involvement: OR 3.388, 95% CI 2.137-5.360, P < 0.001; lateral lymph node involvement: OR 3.468, 95% CI 1.712-7.218, P<0.001; primary nodule only: OR 1.677, 95% CI 0.942-2.926, P = 0.073). The hypocalcemia model showed good discrimination (AUC 0.836, 95% CI 0.810-0.861), as did the hypoparathyroidism model (AUC 0.768, 95% CI 0.752-0.816).

**Conclusions:**

Simple models integrating preoperative calcium, surgical extent, and ultrasound findings effectively predict early postoperative hypocalcemia and hypoparathyroidism in PTC patients undergoing total or subtotal/near-total, which may assist in preoperative risk stratification and guide prophylactic management.

## 1 Introduction

Postoperative hypocalcemia remains one of the most common and clinically significant complications following thyroidectomy for papillary thyroid carcinoma (PTC), with reported incidence rates varying widely from 10% to 45% across clinical studies (1). This wide variability is largely attributed to differences in surgical extent (e.g., total thyroidectomy vs. lobectomy, concurrent neck dissection) and heterogeneous diagnostic criteria for biochemical and symptomatic hypocalcemia (2). Regardless of definition, the clinical impact is substantial: symptomatic hypocalcemia prolongs hospital stay, increases healthcare costs, impairs patient quality of life, and, in severe cases, can lead to life-threatening arrhythmias or laryngospasm (3, 4). Consequently, accurate preoperative identification of patients at heightened risk is essential for implementing targeted preventive strategies and optimizing postoperative management.

The pathogenesis of post-thyroidectomy hypocalcemia is multifactorial, but the central mechanism is parathyroid gland injury—either direct trauma, devascularization, or inadvertent excision during thyroidectomy and concurrent neck dissection (5, 6). This mechanistic link establishes a clear conceptual distinction: hypocalcemia is the clinical manifestation, whereas hypoparathyroidism is its pathophysiological cause (7). The distinction is clinically meaningful—some patients develop hypocalcemia without measurable parathyroid hormone (PTH) deficiency (likely due to hemodilution, albumin binding effects, or other mechanisms) (8), while others with PTH deficiency may remain normocalcemic (due to adequate calcium reserves or compensatory mechanisms) (9, 10). Understanding both outcomes is therefore essential for comprehensive perioperative risk assessment.

Numerous risk factors have been identified for both conditions, including low preoperative calcium levels, female gender, extent of surgery, central and lateral neck dissection, and inadvertent parathyroid gland removal (11–14). However, despite this well-established risk profile, the translation into clinically useful preoperative prediction tools has been limited. Most existing models suffer from one or more limitations: they are derived from small cohorts with limited statistical power (15, 16); they incorporate postoperative variables (such as PTH levels) that are unavailable at the time of surgical decision-making (17, 18); or they include patients undergoing lobectomy—a procedure with minimal hypocalcemia risk—diluting the predictive signal for those undergoing total thyroidectomy (19).

The present study was therefore designed to identify independent preoperative predictors of both early postoperative hypocalcemia and hypoparathyroidism in patients undergoing total or near-total thyroidectomy for PTC. Based on these findings, we developed parsimonious and clinically practical nomograms for individualized risk stratification of each outcome. By assessing both outcomes in parallel, we aimed to provide a more comprehensive framework for understanding and predicting postoperative calcium homeostasis, offering clinicians two complementary tools: one for predicting the clinically actionable endpoint (hypocalcemia) and another for identifying the mechanistic driver (hypoparathyroidism).

## 2 Patients and methods

### 2.1 Study design and patient population

This retrospective study included patients with pathologically confirmed papillary thyroid carcinoma who underwent total or subtotal/near-total thyroidectomy at the First Affiliated Hospital of Xi’an Jiaotong University between January 2014 and February 2021. Inclusion criteria were: (1) age ≥18 years; (2) histologically confirmed PTC; (3) underwent total or subtotal/near-total thyroidectomy; (4) complete preoperative and postoperative laboratory data available. Exclusion criteria were: (1) preoperative hypocalcemia (calcium <2.1 mmol/L); (2) underwent lobectomy; (3) chronic kidney disease affecting calcium metabolism; (4) concomitant parathyroid surgery; (5) missing outcome data.

The study was conducted in accordance with the Declaration of Helsinki and approved by the ethics committee of First Affiliated Hospital of Xi’an Jiaotong University [No.2022-321]. All participants signed an informed consent.

### 2.2 Outcome definition

The primary outcome was early postoperative hypocalcemia, defined as serum calcium level <2.0 mmol/L within 48 hours after surgery or the presence of hypocalcemic symptoms requiring calcium supplementation (including perioral numbness, fingertip paresthesia, muscle cramps, or tetany), irrespective of the measured calcium level.

The secondary outcome was early postoperative hypoparathyroidism, defined as PTH <15 pg/mL accompanied by hypocalcemia within 48 hours after surgery.

Postoperative calcium and vitamin D supplementation was guided by serum calcium levels measured daily. Routine prophylactic supplementation was not administered. Calcium was initiated when serum calcium fell below 2.0 mmol/L, with dosage adjusted according to serial calcium measurements and symptom severity.

### 2.3 Candidate predictors

Candidate predictors were selected based on literature review and clinical expertise, including demographic, biochemical, surgical and ultrasonographic variables.

Biochemical variables: Preoperative variables (measured within 3 days before surgery) included PTH (pg/mL, continuous); thyroid-stimulating hormone (TSH, µIU/mL, continuous); calcium (mmol/L, continuous); and albumin (Alb, g/L, continuous).

Thyroid function status was categorized based on preoperative TSH levels according to the institutional reference range: normal (TSH within normal range), hypothyroid (TSH above normal range), and hyperthyroid (TSH below normal range).

Surgical variables include thyroidectomy extent (total vs. subtotal/near-total), central compartment neck dissection (CCND, unilateral vs. bilateral), lateral neck dissection (LND, not performed vs. performed). Total thyroidectomy was defined as complete removal of all visible thyroid tissue. Subtotal/near-total thyroidectomy was defined as bilateral thyroid resection leaving <1 g of residual thyroid tissue (typically in the region of the recurrent laryngeal nerve entry or paratracheal area) to protect the parathyroid glands and recurrent laryngeal nerve. In our institutional practice, all patients undergoing LND routinely underwent CCND.

Ultrasound risk score was defined based on preoperative ultrasound findings of both the primary nodule and lymph nodes. Primary nodule features included taller-than-wide shape, microcalcifications, and marked hypoechogenicity. Lymph node assessment included the presence of suspicious features (e.g., microcalcifications, cystic change, hyperechogenicity, or rounded shape) in the central (level VI) or lateral (levels II–V) cervical compartments. The composite ultrasound score was categorized as follows: 0 = no suspicious features (primary nodule and lymph nodes both negative); 1 = suspicious primary nodule only; 2 = suspicious central compartment lymph nodes (with or without suspicious primary); and 3 = suspicious lateral compartment lymph nodes (with or without suspicious central or primary features).

### 2.4 Statistical analysis

Continuous variables were summarized as mean ± standard deviation (SD) or median (interquartile range [IQR]). Categorical variables were presented as frequencies and percentages. Group comparisons used Student’s t-test, Wilcoxon rank-sum test, or chi-square test as appropriate.

Univariable logistic regression identified candidate predictors (P < 0.1) for entry into multivariable models. Two separate multivariable models were constructed: one for hypocalcemia and one for hypoparathyroidism.

Model discrimination was assessed using the area under the receiver operating characteristic curve (AUC). Bootstrap internal validation with 500 resamples corrected for optimism. Calibration was evaluated using a calibration plot with bootstrapped confidence intervals. Overall predictive accuracy was quantified by the Brier score. Decision curve analysis (DCA) was performed to evaluate the net benefit of the model across a range of threshold probabilities. Optimal thresholds were determined based on the Youden index.

All statistical analyses were performed using R software version 4.5.1. A two-sided p<0.05 was considered statistically significant.

## 3 Results

### 3.1 Study population characteristics

A total of 1036 patients were included in the final analysis, of whom 325 (31.4%) developed early postoperative hypocalcemia, and 228 (22.0%) met the criteria for early hypoparathyroidism. **Table 1** presents the baseline characteristics.

**Table 1:** Baseline characteristics of patients stratified by hypocalcemia and hypoparathyroidism status.

Characteristic	Total (N = 1036)	Hypocalcemia			Hypoparathyroidism		
		No (N = 711)	Yes (N = 325)	P-value	No (N = 808)	Yes (N = 228)	P-value
Demographics							
Age (years), mean ± SD	43.79 ± 12.66	44.07 ± 12.51	43.19 ± 12.98	0.300	44.16 ± 12.45)	42.47 ± 13.30	0.075
Sex, n (%)				0.266			0.177
Male	303 (29.2)	216 (30.4)	87 (26.8)		245 (30.3)	58 (25.4)	
Female	733 (70.8)	495 (69.6)	238 (73.2)		563 (69.7)	170 (74.6)	
Biochemical variables							
Preoperative PTH (pg/mL), mean ± SD	49.39 ± 20.00	48.90 ± 18.40	50.48 ± 23.13	0.237	49.62 ± 19.88	48.58 ± 20.45	0.485
Preoperative TSH (µIU/mL), median [IQR]	1.67 [1.03–2.66]	1.61 [1.02–2.42]	1.95 [1.11–2.96]	0.009	1.63 [1.02–2.49]	1.95 [1.14–2.92]	0.009
Preoperative calcium (mmol/L), mean ± SD	2.25 ± 0.12	2.29 ± 0.13	2.18 ± 0.06	<0.001	2.27 ± 0.13	2.19 ± 0.06	<0.001
Preoperative Alb (g/L), mean ± SD	43.14 ± 3.95	43.53 ± 3.89	42.29 ± 3.96	0.001	43.36 ± 3.90	42.37 ± 4.06	0.001
Thyroid Function, n (%)				0.032			0.088
Normal	933 (90.1)	652 (91.7)	281 (86.5)		736 (91.1)	197 (86.4)	
Hypothyroid	86 (8.3)	49 (6.9)	37 (11.4)		59 (7.3)	27 (11.8)	
Hyperthyroid	17 (1.6)	10 (1.4)	7 (2.2)		13 (1.6)	4 (1.8)	
Surgical variables							
Thyroidectomy, n (%)				0.012			0.105
Total	1009 (97.4)	686 (96.5)	323 (99.4)		783 (96.9)	226 (99.1)	
Subtotal/Near-total	27 (2.6)	25 (3.5)	2 (0.6)		25 (3.1)	2 (0.9)	
Central compartment dissection, n (%)				0.051			0.049
Unilateral	677 (65.3)	479 (67.4)	198 (60.9)		541 (67.0)	136 (59.6)	
Bilateral	359 (34.7)	232 (32.6)	127 (39.1)		267 (33.0)	92 (40.4)	
Lateral neck dissection, n (%)				<0.001			<0.001
Not performed	802 (77.4)	621 (87.3)	181 (55.7)		669 (82.8)	133 (58.3)	
Performed	234 (22.6)	90 (12.7)	144 (44.3)		139 (17.2)	95 (41.7)	
Ultrasound							
Ultrasound risk score, n (%)				<0.001			<0.001
No suspicious	631 (60.9)	524 (73.7)	107 (32.9)		556 (68.8)	75 (32.9)	
Primary nodule only	103 (9.9)	56 (7.9)	47 (14.5)		74 (9.2)	29 (12.7)	
Central LN involvement	147 (14.2)	86 (12.1)	61 (18.8)		100 (12.4)	47 (20.6)	
Lateral LN involvement	155 (15.0)	45 (6.3)	110 (33.8)		78 (9.7)	77 (33.8)	

SD, standard deviation; IQR, interquartile range; Alb, albumin; LN, lymph node; PTH, parathyroid hormone; TSH, thyroid-stimulating hormone.

Patients with early hypocalcemia had significantly lower preoperative calcium (2.18 ± 0.06 vs. 2.29 ± 0.13 mmol/L, P < 0.001), lower albumin (42.29 ± 3.96 vs. 43.53 ± 3.89 g/L, P < 0.001), higher TSH (1.95 [1.11–2.96] vs. 1.61 [1.02–2.42] µIU/mL, P = 0.009), higher rates of total thyroidectomy (99.4% vs. 96.5%, P = 0.012), higher rates of LND (44.3% vs. 12.7%, P < 0.001), and a higher proportion of patients with ultrasound-detected lymph node involvement (52.6% vs. 18.4%, P < 0.001).

Similarly, patients with early hypoparathyroidism exhibited significantly lower preoperative calcium (2.19 ± 0.06 vs. 2.27 ± 0.13 mmol/L, P < 0.001), lower albumin (42.37 ± 4.06 vs. 43.36 ± 3.90 g/L, P < 0.001), higher TSH (1.95 [1.14–2.92] vs. 1.63 [1.02–2.49] µIU/mL, P = 0.009), higher rates of LND (41.7% vs. 17.2%, P < 0.001), and a higher proportion of patients with ultrasound-detected lymph node involvement (54.4% vs. 22.1%, P < 0.001). Besides, patients with early hypoparathyroidism had a higher rate of bilateral CCND compared with those without (40.4% vs. 33.0%, P = 0.049).

### 3.2 Predictors of early postoperative hypocalcemia

Univariable logistic regression was performed to screen candidate predictors (**Table 2**), and variables with P < 0.1 were entered into multivariable model. Multivariable analysis identified independent preoperative predictors of early postoperative hypocalcemia, including preoperative calcium, thyroidectomy extent, LND, ultrasound risk score, and thyroid function (**Table 3**).

**Table 2:** Univariate logistic regression analysis for early postoperative hypocalcemia and hypoparathyroidism.

Characteristic	Hypocalcemia		Hypoparathyroidism	
	OR (95% CI)	P-value	OR (95% CI)	P-value
Demographics				
Age (per year)	0.995 (0.984-1.005)	0.300	0.989 (0.978-1.001)	0.076
Sex				
Female vs. Male	1.194 (0.891-1.600)	0.236	1.275 (0.914-1.781)	0.153
Biochemical variables				
Preoperative PTH (per 1 pg/mL)	1.004 (0.997-1.010)	0.238	0.997 (0.990-1.005)	0.485
Preoperative TSH (per 1 µIU/mL)	1.088 (1.019-1.161)	0.012	1.091 (1.019-1.169)	0.012
Preoperative calcium (per 0.1 mmol/L)	0.665 (0.609-0.730)	<0.001	0.845 (0.747-0.956)	<0.001
Preoperative Alb (per 1 g/L)	0.922 (0.890-0.954)	<0.001	0.937 (0.902-0.974)	<0.001
Thyroid Function (vs. Normal)				
Hypothyroid	1.752 (1.118-2.746)	0.014	1.710 (1.056-2.768)	0.029
Hyperthyroid	1.624 (0.612-4.310)	0.330	1.150 (0.371-3.564)	0.809
Surgical variables				
Thyroidectomy				
Subtotal/Near-total vs. Total	0.170 (0.040-0.721)	0.016	0.277 (0.065-1.177)	0.082
Central compartment dissection				
Bilateral vs. Unilateral	1.324 (1.008-1.739)	0.043	1.371 (1.013-1.855)	0.041
Lateral neck dissection				
Performed vs. Not	5.490 (4.022-7.493)	<0.001	3.438 (2.495-4.737)	<0.001
Ultrasound				
Ultrasound risk score				
No suspicious (0)	Ref		Ref	
Primary nodule only (1)	4.110 (2.647-6.382)	<0.001	2.905 (1.775-4.754)	<0.001
Central LN involvement (2)	3.474 (2.356-5.122)	<0.001	3.484 (2.284-5.315)	<0.001
Lateral LN involvement (3)	11.971 (7.989-17.937)	<0.001	7.318 (4.922-10.880)	<0.001

Data are presented as odds ratio (OR) with 95% confidence interval (CI). ORs for continuous variables represent the change in risk per unit increase.

**Table 3:** Multivariate logistic regression analysis for early postoperative hypocalcemia and hypoparathyroidism.

Characteristic	Hypocalcemia		Hypoparathyroidism	
	OR (95% CI)	P-value	OR (95% CI)	P-value
Biochemical variables				
Preoperative TSH (per 1 µIU/mL)	1.024 (0.918-1.151)	0.678	1.045 (0.936-1.172)	0.438
Preoperative calcium (per 0.1 mmol/L)	0.298 (0.234-0.374)	<0.001	0.417 (0.330-0.519)	<0.001
Preoperative Alb (per 1 g/L)	1.002 (0.957-1.048)	0.946	1.002 (0.958-1.048)	0.924
Thyroid Function (vs. Normal)				
Hypothyroid	1.082 (0.485-2.373)	0.845	1.004 (0.449-2.183)	0.992
Hyperthyroid	3.405 (1.010-10.914)	0.041	1.941 (0.492-6.305)	0.298
Surgical variables				
Thyroidectomy				
Subtotal/Near-total vs. Total	0.175 (0.025-0.709)	0.033	0.341 (0.052-1.278)	0.168
Central compartment dissection				
Bilateral vs. Unilateral	0.869 (0.600-1.251)	0.454	0.996 (0.690-1.431)	0.983
Lateral neck dissection				
Performed vs. Not	3.197 (1.742-5.852)	<0.001	1.494 (0.766-2.828)	0.227
Ultrasound				
Ultrasound risk score				
No suspicious (0)	Ref		Ref	
Primary nodule only (1)	1.899 (1.119-3.198)	0.016	1.677 (0.942-2.926)	0.073
Central LN involvement (2)	4.013 (2.559-6.323)	<0.001	3.388 (2.137-5.360)	<0.001
Lateral LN involvement (3)	3.387 (1.700-6.825)	<0.001	3.468 (1.712-7.218)	<0.001

Higher preoperative calcium was associated with decreased risk of early hypocalcemia (OR 0.298 per 0.1 mmol/L, 95% CI 0.234–0.374, P < 0.001), corresponding to an approximately 70.2% risk reduction for each 0.1 mmol/L increase. Subtotal/near-total thyroidectomy was associated with a lower risk compared with total thyroidectomy (OR 0.175, 95% CI 0.025–0.709, P = 0.033). LND was associated with a markedly increased risk (OR 3.197, 95% CI 1.742–5.852, P < 0.001). For ultrasound risk score, compared with no suspicion, primary nodule only (OR 1.899, 95% CI 1.119–3.198, P = 0.016), central lymph node involvement (OR 4.013, 95% CI 2.559–6.323, P < 0.001), and lateral lymph node involvement (OR 3.387, 95% CI 1.700–6.825, P < 0.001) were all associated with increased risk. Hyperthyroid status was also associated with increased risk (OR 3.405, 95% CI 1.010–10.914, P = 0.041).

### 3.3 Predictors of early postoperative hypoparathyroidism

Univariable and multivariable logistic regression analyses were also performed for early hypoparathyroidism (**Table 2**). Multivariable analysis identified preoperative calcium and ultrasound risk score as independent predictors (**Table 3**).

Higher preoperative calcium was protective against early hypoparathyroidism (per 0.1 mmol/L: OR 0.417, 95% CI 0.330–0.519, P < 0.001). For ultrasound risk score, compared with no suspicion, central lymph node involvement (OR 3.388, 95% CI 2.137–5.360, P < 0.001) and lateral lymph node involvement (OR 3.468, 95% CI 1.712–7.218, P < 0.001) were associated with increased risk; primary nodule only did not reach statistical significance (OR 1.677, 95% CI 0.942–2.926, P = 0.073).

### 3.4 Subgroup analysis: effect of LND in patients undergoing CCND

To further characterize the relationship between surgical extent and postoperative outcomes, we stratified patients by both CCND extent (unilateral vs. bilateral) and LND status. The incidence of hypocalcemia increased progressively with surgical extent: unilateral CCND alone (23.7%), bilateral CCND alone (19.7%), unilateral CCND with LND (58.3%), and bilateral CCND with LND (64.3%) (P for trend < 0.001). A similar gradient was observed for hypoparathyroidism: 17.4%, 14.6%, 34.3%, and 46.0%, respectively (P for trend < 0.001).

On multivariable analysis using unilateral CCND alone as the reference, LND remained a significant independent predictor of early hypocalcemia (unilateral CCND with LND: OR 2.65, 95% CI 1.37-5.14, P = 0.004; bilateral CCND with LND: OR 3.44, 95% CI 1.73-6.86, P < 0.001). For early hypoparathyroidism, the association was directionally consistent but did not reach statistical significance (bilateral CCND with LND: OR 1.90, 95% CI 0.93-3.79, P = 0.071). These findings confirm that LND confers additional risk beyond CCND alone, with a particularly pronounced effect on hypocalcemia. Detailed outcomes by surgical extent are presented in **Supplementary Table 1**.

### 3.5 Nomogram development

Based on the final multivariable models, two nomograms were constructed to provide visual tools for predicting individual risk. The hypocalcemia nomogram incorporated preoperative calcium, thyroidectomy extent, LND, ultrasound risk score, and thyroid function (**Figure 1A**), while the hypoparathyroidism nomogram included preoperative calcium and ultrasound risk score (**Figure 1B**).

**Figure 1:**
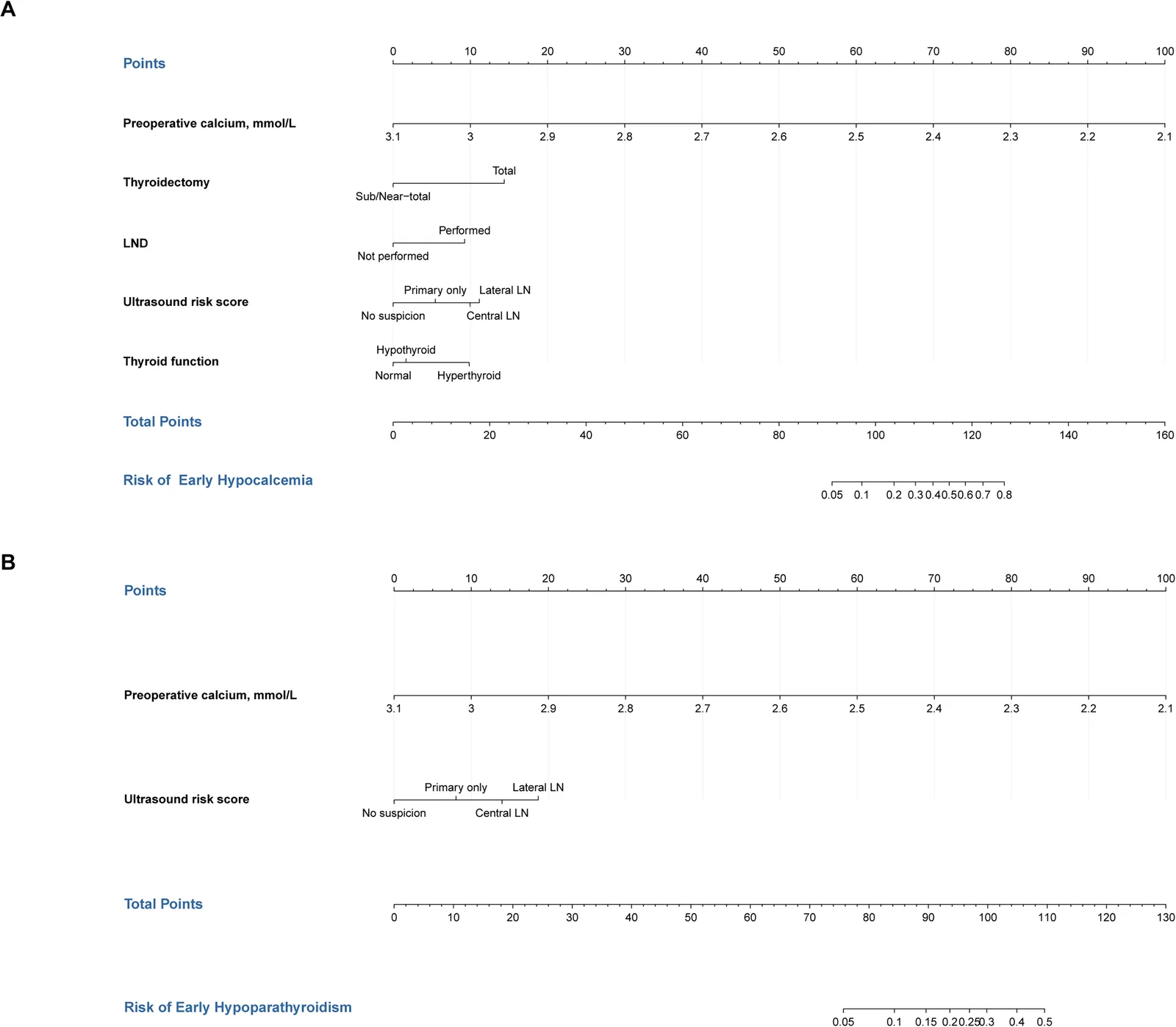
Nomograms for predicting early postoperative outcomes. **(A)** Nomogram for predicting early postoperative hypocalcemia, incorporating preoperative calcium, thyroidectomy extent, lateral neck dissection, ultrasound risk score and thyroid function. **(B)** Nomogram for predicting early postoperative hypoparathyroidism, incorporating preoperative calcium and ultrasound risk score.

To use the nomograms, locate each variable value on its corresponding axis, draw a vertical line to the “Points” axis to obtain points values, sum all points, and project the total points to the “Risk” axis to obtain the predicted probability of the outcome.

### 3.6 Nomogram performance

The hypocalcemia nomogram demonstrated good discrimination with an apparent AUC of 0.836 (95% CI: 0.810-0.861) (**Figure 2**). Bootstrap internal validation (500 resamples) confirmed the robustness of the model, with the corrected AUC showing minimal shrinkage from the apparent AUC (0.836 vs. 0.836). The Brier score was 0.144, suggesting acceptable predictive accuracy. Similarly, the hypoparathyroidism nomogram showed an apparent AUC of 0.784 (95% CI: 0.752-0.816) (**Figure 2**), with a bootstrap-corrected AUC of 0.785 (95% CI: 0.749-0.815) and a Brier score of 0.140. The DeLong test revealed a statistically significant difference in AUC between the two models (P = 0.013).

**Figure 2:**
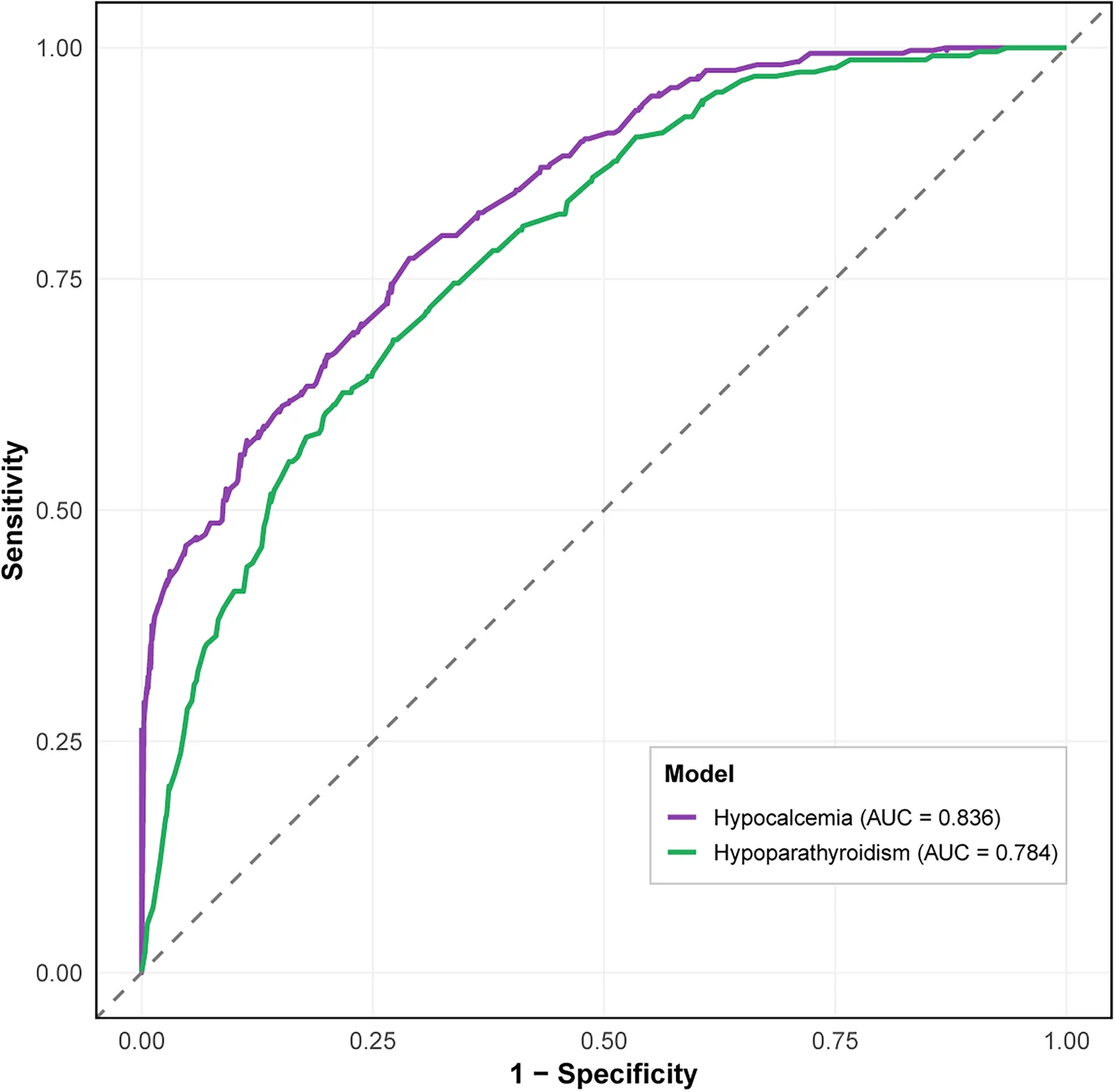
Receiver operating characteristic (ROC) curves of the prediction models. The early hypocalcemia model (purple) demonstrated an AUC of 0.836 (95% CI: 0.810–0.861), and the early hypoparathyroidism model (green) demonstrated an AUC of 0.784 (95% CI: 0.752–0.816). The diagonal dashed line represents the reference line of no discriminative ability (AUC = 0.5).

Calibration curves demonstrated good agreement between predicted probabilities and observed outcomes for both models (**Figure 3**), with predicted probabilities closely matching observed frequencies across the clinically relevant risk range. DCA demonstrated that both nomograms provided positive net benefit across a range of clinically relevant threshold probabilities, outperforming both “treat-all” and “treat-none” strategies (**Figures 4A, B**), confirming their clinical utility for guiding preventive decision-making.

**Figure 3:**
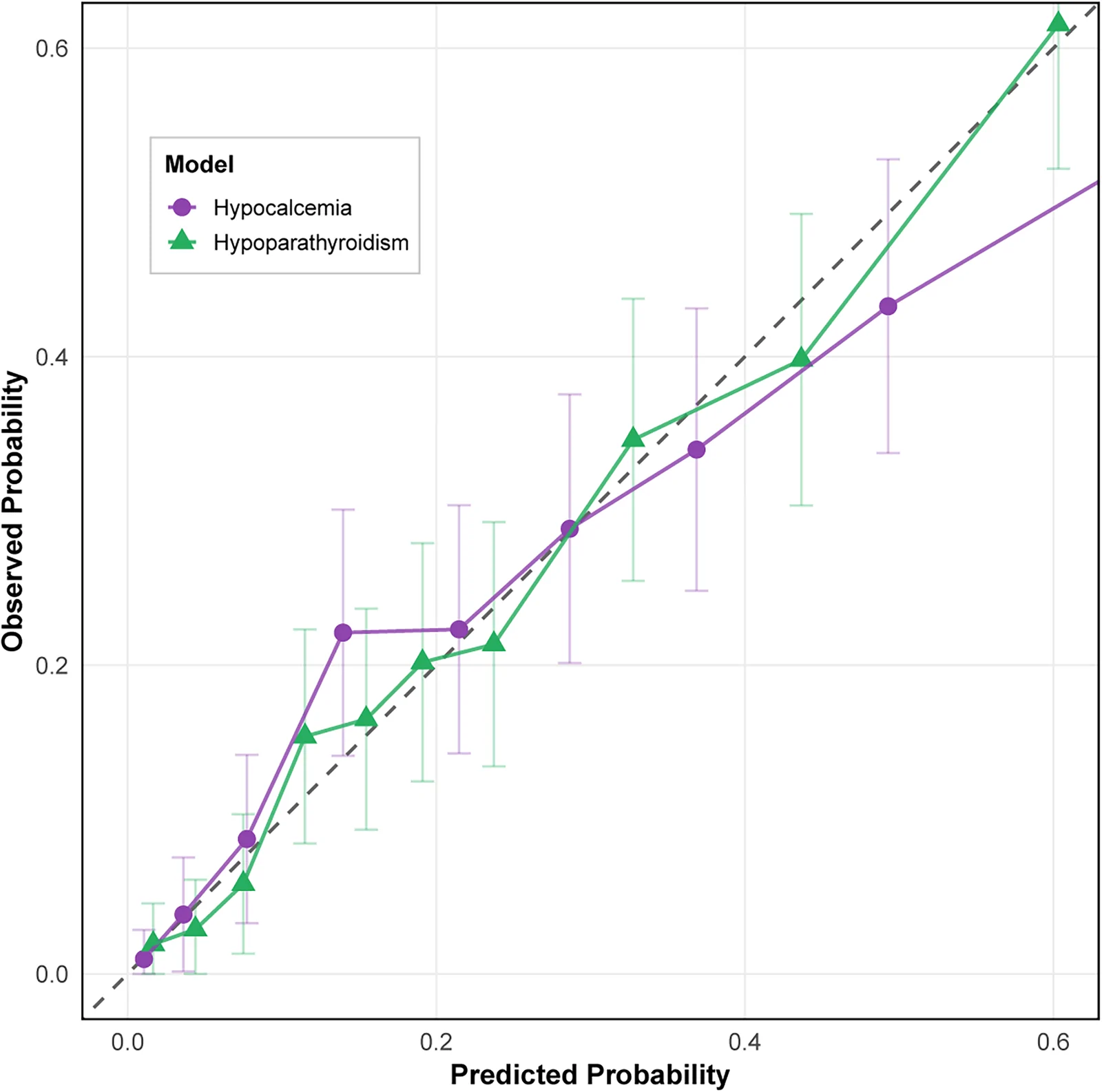
Calibration plot of the prediction models. Calibration plot for the hypocalcemia model (purple) and calibration plot for the hypoparathyroidism model (green) showed good agreement between predicted probabilities and observed outcomes. The diagonal dashed line indicates perfect calibration (ideal prediction).

**Figure 4:**
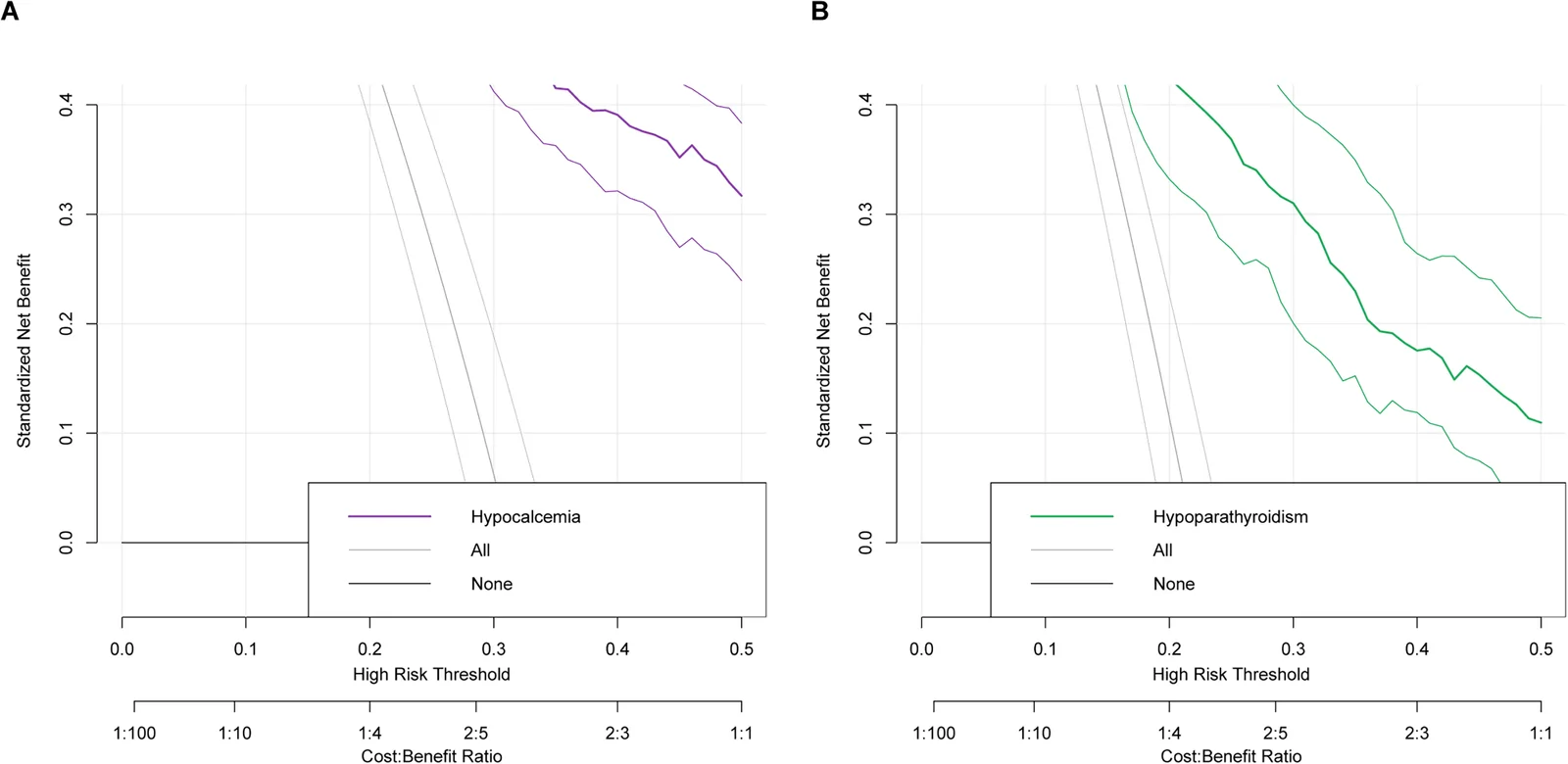
Decision curve analysis (DCA) for the prediction models. **(A)** DCA for the hypocalcemia model. **(B)** DCA for the hypoparathyroidism model. The solid line represents the net benefit of the prediction model. The gray dashed line represents the “treat-all” strategy, and the black dashed line represents the “treat-none” strategy.

At the optimal threshold of 0.30 (determined by the Youden index), the hypocalcemia model achieved a sensitivity of 77.2%, specificity of 71.0%, PPV of 54.9%, and NPV of 87.2% (**Table 4**). For the hypoparathyroidism model, the optimal threshold was 0.23, with a sensitivity of 68.4%, specificity of 72.8%, PPV of 41.5%, and NPV of 89.1%. The high NPVs indicate that both models are particularly useful for ruling out complications in low-risk patients.

**Table 4:** Model performance comparison.

Metric	Hypocalcemia Model	Hypoparathyroidism Model
Variables	Preoperative calcium + Thyroidectomy + Lateral neck dissection + Ultrasound risk score + Thyroid function	Preoperative calcium + Ultrasound risk score
AUC	0.836	0.784
95% CI	0.810-0.861	0.752-0.816
Optimal Threshold	0.30	0.23
Sensitivity (%)	77.2	68.4
Specificity (%)	71.0	72.8
PPV (%)	54.9	41.5
NPV (%)	87.2	89.1
Accuracy (%)	73	71.8
Brier Score	0.144	0.140

AUC, area under the curve; PPV, positive predictive value; NPV, negative predictive value; DeLong test P = 0.013 for AUC comparison between models.

## 4 Discussion

In this large retrospective study of 1036 patients who underwent total or near-total thyroidectomy for PTC, we developed and validated two complementary prediction models—one for early postoperative hypocalcemia and one for hypoparathyroidism. The early hypocalcemia model incorporated preoperative calcium, thyroidectomy extent, LND, ultrasound risk score, and thyroid function (AUC 0.836, 95% CI 0.810-0.861); the early hypoparathyroidism model incorporated preoperative calcium and ultrasound risk score (AUC 0.784, 95% CI 0.752–0.816). Both models demonstrated good discrimination, acceptable calibration, and positive net benefit on DCA. The most important finding of our study is that early hypocalcemia and hypoparathyroidism, while closely related, are predicted by distinctly different sets of preoperative variables. Understanding both outcomes in parallel provides a more complete picture of early postoperative calcium homeostasis than either outcome alone.

Preoperative calcium was the strongest predictor for both outcomes. Each 0.1 mmol/L increase in preoperative calcium reduced the odds of hypocalcemia by 70.2% (OR 0.298) and hypoparathyroidism by 58.3% (OR 0.417). This dose-response relationship aligns with the concept of parathyroid functional reserve: patients with higher baseline calcium have greater physiological capacity to withstand transient parathyroid insufficiency following surgical stress (20, 21). Furthermore, randomized trials found that short-term preoperative oral calcium supplementation could effectively reduce the incidence of postoperative hypocalcemia in patients undergoing bilateral thyroidectomy, underscoring the clinical relevance of preoperative calcium assessment (22, 23). The differential magnitude of the effect—calcium is more strongly protective against hypocalcemia than against hypoparathyroidism—is noteworthy. This suggests that while low preoperative calcium is a marker of limited parathyroid reserve (hence its association with both outcomes), the protective effect against clinical hypocalcemia is amplified because patients with higher baseline calcium can tolerate a greater degree of PTH decline before becoming overtly hypocalcemic. This observation underscores the clinical utility of preoperative calcium as a risk stratification tool: patients with low-normal preoperative calcium (e.g., 2.1–2.2 mmol/L) warrant closer postoperative monitoring even if PTH levels are not profoundly depressed.

Our subgroup analysis provides the clearest evidence of a dose-response relationship between surgical extent and postoperative risk. The incidence of early hypocalcemia increased progressively from unilateral CCND alone (23.7%) to unilateral CCND with LND (58.3%) to bilateral CCND with LND (64.3%), with a similar gradient for hypoparathyroidism (17.4%, 34.3%, and 46.0%, respectively) (P for trend < 0.001 for both outcomes). This gradient validates the clinical relevance of preoperative ultrasound assessment of lymph node involvement—precisely the variable we incorporated into our refined risk score. Two observations from this analysis merit discussion. First, LND was a strong predictor of early hypocalcemia (OR 3.197, P < 0.001) but not of early hypoparathyroidism (OR 1.494, P = 0.227). This discrepancy suggests that LND contributes to hypocalcemia through mechanisms beyond PTH deficiency, including hemodilution from increased intraoperative fluid administration, transient suppression of parathyroid function not captured by a single postoperative PTH measurement, or concurrent magnesium disturbances (24–27). This reinforces the clinical message that patients undergoing LND require systematic postoperative calcium monitoring regardless of whether PTH criteria for hypoparathyroidism are met. Second, bilateral CCND alone appeared protective compared with unilateral CCND alone (OR 0.72, P = 0.150), though this did not reach statistical significance. This likely reflects selection bias rather than a true protective effect: in high-volume centers, bilateral CCND is more often performed by experienced surgeons who employ meticulous parathyroid preservation techniques. It does not diminish the central message that LND—the most extensive procedure—confers the highest risk.

Subtotal/near-total thyroidectomy was protective against early hypocalcemia (OR 3.197, P < 0.001) but not hypoparathyroidism (OR 0.341, P = 0.168). This aligns with a randomized controlled trial comparing near-total versus total thyroidectomy in Graves’ disease, which found no significant difference in transient hypoparathyroidism rates between the two procedures (19% vs. 21%, P = 0.84) (28). The key determinant remains intraoperative parathyroid gland preservation rather than the extent of resection alone (29). Notably, total thyroidectomy patients had a higher proportion of LND (23.1% vs. 3.75%), reflecting that more extensive surgery was performed for more advanced disease. This complexity underscores the need for comprehensive preoperative risk assessment that integrates both biochemical and surgical factors, as the extent of resection may serve as a proxy for disease burden rather than an independent driver of parathyroid dysfunction (30).

The refined ultrasound risk score—incorporating both primary nodule features and lymph node status—demonstrated a clear hierarchical gradient. Compared with no suspicion, primary nodule only (OR 1.899, P = 0.016), central lymph node involvement (OR 4.013, P < 0.001), and lateral lymph node involvement (OR 3.387, P < 0.001) showed progressively increasing early hypocalcemia risk. A similar gradient was observed for early hypoparathyroidism. This finding extends the utility of preoperative ultrasound beyond oncological risk assessment to functional risk prediction. The hierarchical nature of this scoring system—with progressively higher ORs from primary-only to central to lateral lymph node involvement—supports the biological plausibility that more extensive disease requiring more extensive dissection increases the risk of parathyroid injury (31). For clinicians, this means that routine preoperative ultrasound can simultaneously serve two purposes: oncological staging and functional risk stratification, without additional cost or testing.

In contrast to the well-established predictive value of postoperative PTH for hypocalcemia (32), preoperative PTH was not identified as a significant predictor in our univariable analysis (P = 0.238 for hypocalcemia; P = 0.485 for hypoparathyroidism). This finding is consistent with a recent study of 295 patients demonstrating that day 1 postoperative PTH is equivalent to (if not better than) the perioperative PTH drop in predicting short- and long-term hypoparathyroidism, with authors explicitly concluding that “preoperative PTH measurements may not be needed in the detection and/or management of postoperative hypoparathyroidism after thyroid surgery” (33). Preoperative PTH reflects baseline parathyroid function, whereas postoperative hypocalcemia and hypoparathyroidism are primarily determined by intraoperative parathyroid gland injury—a dynamic process that preoperative PTH cannot capture (34). In our cohort, the strong predictive power of surgical variables supports this interpretation: it is the surgical insult, not the preoperative hormonal setpoint, that ultimately determines postoperative parathyroid function. Compared with existing models that often require postoperative PTH measurements or pathological findings (35–38), our model offers the advantage of being entirely preoperative and intraoperative, allowing risk stratification in time to guide preoperative counseling, nutritional optimization, and surgical planning.

This study has several notable strengths. The large, well-characterized cohort of 1,036 patients substantially exceeds the sample sizes of most previous prediction model studies (14, 39, 40), providing robust statistical power. The dual-outcome design—analyzing both hypocalcemia and hypoparathyroidism in parallel—allowed comparison between the clinical manifestation and its underlying mechanism. The refined ultrasound risk score, incorporating both primary nodule features and lymph node status, provides a more comprehensive preoperative assessment. While we focused on early postoperative risk prediction, future studies with extended follow-up are warranted to determine whether the preoperative predictors identified in our models also correlate with long-term parathyroid outcomes. Finally, our models rely exclusively on routinely available preoperative variables, enhancing clinical translatability without additional cost or specialized testing.

Several limitations should be acknowledged. First, this is a retrospective, single-center study, which may limit generalizability. Second, prospective multicenter external validation is urgently needed to confirm the generalizability of our models. Third, we lacked detailed intraoperative data on parathyroid gland preservation or auto-transplantation. Fourth, our definition of hypoparathyroidism was time-bound to the early postoperative period (48 hours) and did not distinguish between transient and permanent forms. Fifth, preoperative vitamin D levels were not routinely available for the majority of patients, and therefore could not be included in the analysis. Finally, our models were developed in PTC patients undergoing total or near-total thyroidectomy; their applicability to other thyroid pathologies or less extensive procedures remains to be determined.

## 5 Conclusions

We developed and internally validated two simple, clinically practical prediction models for early postoperative hypocalcemia and hypoparathyroidism using only preoperatively available variables in patients undergoing total or near-total thyroidectomy for PTC. The differential predictors for the two outcomes provide mechanistic insight and support the conceptual distinction between clinical hypocalcemia and its pathophysiological driver.

## Data Availability

The raw data supporting the conclusions of this article will be made available by the authors, without undue reservation.
