# Supplementary material for: Prediction of early postoperative hypocalcemia and hypoparathyroidism after thyroidectomy for papillary thyroid carcinoma: dual-outcome prediction models
Source: Front Endocrinol (Lausanne). 2026 Sep 30;17:1887201. doi: 10.3389/fendo.2026.1887201 (PMC13661344; doi:10.3389/fendo.2026.1887201)
Supplement: Supplementary file 1 [file Table1.docx]

**Supplementary Table 1.** Surgical extent and postoperative outcomes

| **Surgery Extent** | **N** | **Hypocalcemia, n (%)** | **Hypoparathyroidism, n (%)** | **Adjusted OR for Hypocalcemia (95% CI) *** | **Adjusted OR for Hypoparathyroidism (95% CI) *** |
| --- | --- | --- | --- | --- | --- |
| **Unilateral CCND only** | 569 | 135 (23.7) | 99 (17.4) | 1 (ref) | 1 (ref) |
| **Bilateral CCND only** | 233 | 46 (19.7) | 34 (14.6) | 0.72 (0.45-1.12) | 0.72 (0.44-1.15) |
| **Unilateral CCND + LND** | 108 | 63 (58.3) | 37 (34.3) | **2.65 (1.37-5.14)** | 1.14 (0.55-2.28) |
| **Bilateral CCND + LND** | 126 | 81 (64.3) | 58 (46.0) | **3.44 (1.73-6.86)** | 1.90 (0.93-3.79) |
| **Unilateral CCND only** | 569 | 135 (23.7) | 99 (17.4) | 1 (ref) | 1 (ref) |
| **Bilateral CCND only** | 233 | 46 (19.7) | 34 (14.6) | 0.72 (0.45-1.12) | 0.72 (0.44-1.15) |
| **P for trend** |  | **<0.001** | **<0.001** |  |  |

*Adjusted for preoperative calcium, albumin, thyroidectomy extent, and ultrasound risk score. Values in bold indicate statistical significance (P < 0.05).
